# Reproductive Biology and Population Structure of the Endangered Species *Sonneratia ovata* Backer

**DOI:** 10.3390/biology14111580

**Published:** 2025-11-12

**Authors:** Shi-Quan Wang, Feiyan Ren

**Affiliations:** 1 School of Biology and Food Engineering, Fuyang Normal University, Fuyang 236037, China; 2 College of Life Sciences, Hainan Normal University, Haikou 571158, China

**Keywords:** genetic diversity, mating system, population structure, *Sonneratia ovata*, SSR

## Abstract

*Sonneratia ovata* is an important tree species for ornamental, economic, ecological, and medicinal value and is identified as an endangered species. There are very few studies on the reproductive traits, genetic diversity, and population structure of *S. ovata*. Therefore, it is urgent to accurately understand its genetic background and reproductive status in order to better conserve and manage *S. ovata*. *S. ovata* has a mixed mating system, is partially self-compatible and needs pollinators, according to the outcrossing index, pollen–ovule ratio, pollination treatment results and outcrossing rate. Natural populations maintained high outcrossing coupled with inbreeding and low genetic diversity (*H*_e_ = 0.215), and the population DC was regarded as the center of genetic diversity. The Mantel test showed that there existed a positive correlation between geographic and genetic distance among populations, which was in line with the IBD model. Molecular variance was largely confined to within-population differences (75.4%), while inter-population differences accounted for 24.6%. Structure and PCoA analysis supported the UPGMA cluster. This study is the first to investigate reproductive traits, genetic diversity, and population structure through SSR. The results provide a scientific basis for cross breeding, conservation, and management of this species. In future, it is necessary to increase relevant research (human, environment, habitat factors, etc.) to better protect and utilize this species.

## Simple Summary

*Sonneratia ovata* is a mangrove species with important ornamental, economic, ecological, and medicinal value that is listed as an endangered species. However, there are few studies on the reproductive traits, genetic diversity, and population structure of *S. ovata.* People could not accurately understand its genetic background and reproductive status, and therefore could not conserve and manage it better. In order to understand the genetic background and reproductive status of *S. ovata*, pertinent studies were carried out through pollination, mating system, and SSR method. *S. ovata* has a mixed mating system, is partially self-compatible and needs pollinators, according to the outcrossing index, pollen–ovule ratio, pollination treatment results and outcrossing rate. The genetic diversity (*H*_e_ = 0.215) of populations was low, and the population DC was regarded as the center of genetic diversity. The Mantel test showed that there existed a positive correlation between geographic and genetic distance (*r*^2^ = 0.4841 *p =* 0.05) among populations, which was in line with the IBD model. Molecular variance mainly existed within populations (75.4%), while only 24.6% existed among populations (*p* < 0.001). Structure and PCoA analysis supported the UPGMA cluster. This study is the first to investigate reproductive traits, genetic diversity, and population structure through SSR. The results provide a scientific basis for cross breeding, conservation, and management of this species.

## 1. Introduction

Mangrove forests are one of the most important ecosystems on earth [1,2]. Mangroves offer a large number of goods and services to society in addition to their important ecological roles [3]. Mangroves provide goods such as timber, fuelwood, charcoal, and medicines [4].

Genus *Sonneratia* (Lythraceae) is a major and typical genus in mangrove forests. Species of *Sonneratia* belong to non-viviparous mangrove plants, but they have strong vitality and adaptability to intertidal environments and important ecological and economic value [5]. With the continuous degradation and destruction of mangrove forests worldwide, there is an urgent need to investigate basic genetic information in order to develop effective conservation strategies.

*Sonneratia ovata* Backer, belonging to the genus *Sonneratia* Linn. (Lythraceae), grows at the coast, and is resistant to wind and waves, tolerates barren and saline soils, and protects the coastline. The fruits are edible and possess significant industrial potential, particularly in the soft drinks industry. In addition, several chemical compounds, including triterpenes and steroids, have been isolated from the branches of *S. ovata*. These compounds exhibit strong bioactivities, such as antitumor and hemolysis properties, making them valuable for new drug discovery [6,7]. Thus, *S. ovata* has significant ecological and economic values, serving roles in coastal defense, industrial applications, food production, medicine, and ornamental use.

Due to the improper development and utilization of aquaculture, tourism, raising poultry, etc., habitats have been seriously destroyed, fragmented, and deteriorated. The range of distribution has continuously contracted, and the population and individual numbers of *S*. *ovata* have been gradually reduced. The natural regeneration conditions have deteriorated, resulting in difficulties in natural regeneration and population expansion. The natural distribution range of *S. ovata* is narrow and sporadic in China, and only distributed in Qinglan Provincial Nature Reserve in Wenchang of China. According to previous studies, it has been identified as an endangered species (EN) [8] or a critically endangered species (CR) [9].

Morphological and physiological traits have been used to assess diversity. However, the information was very limited for *S. ovata* because those features are unstable in natural environmental conditions. Researchers have employed several molecular markers, including inter simple sequence repeats (ISSRs), arbitrarily primed PCR (AP-PCR), and random amplified polymorphic DNA (RAPD), to investigate the genetic relationships among species [10,11,12] and genetic diversity of some species in *Sonneratia* [13,14,15,16,17,18]. Compared with the above molecular markers, SSR has more advantages (co-dominant, highly polymorphic, highly reproducible, and highly reliable) [19].

Previous research on *S. ovata* has only focused on genetic relationships among species [11,12] and genetic diversity using ISSR markers [15]. However, no one has used SSR markers for measuring genetic diversity, population structure, and mating systems. This knowledge gap limits conservation and management strategies for this endangered species.

Some studies on the pollination biology of several species of *Sonneratia* were conducted, and they identified animals such as insects, birds, and mammals as pollinators [20,21,22]. However, there is a lack of study on the pollination and mating systems of *S. ovata*, which affects the effective conservation of *S. ovata*.

In this study, we aim to (1) test pollen viability and stigma receptivity; (2) calculate outcrossing index (OCI) and pollen–ovule ratio (P/O); (3) analyze the results of pollination treatments and mating systems; (4) assess genetic diversity and population structure; (5) analyze gene flow and genetic differentiation; and (6) propose conservation implications and management strategies. Our findings will contribute to the conservation, management, and sustainable utilization of this endangered mangrove species.

## 2. Materials and Methods

### 2.1. Pollen Viability and Stigma Receptivity Test

Pollen viability was tested using the sucrose boric-acid germination method [23] on days 1–5 after flowering, and was assessed by counting more than 500 pollen grains. Stigma receptivity was tested using the benzidine-hydrogen peroxide method [24].

### 2.2. Mating System Estimation

#### 2.2.1. Outcrossing Index and Pollen–Ovule Ratio Calculation

The outcrossing index (OCI) was estimated based on flower diameter, floral traits, and flowering behavior following the criteria established by Dafni [24]. These traits were measured from five randomly selected flowers per tree across all sampled populations. We collected mature indehiscent flower buds before anthesis and stored them in FAA, then calculated the pollen–ovule ratio (P/O) [25,26].

#### 2.2.2. Pollination Treatments

Every pollination treatment was executed on 60 flowers of trees chosen randomly (6 trees, each with 10 flowers). Seven pollination treatment groups were used to decide mating system type [26]. Under normal circumstances, the fruit grew slowly, and one month after pollination, the number of grown fruits would be counted. During this period, some could not grow or would shrink. The fruits were harvested separately when ripe in November 2021.

### 2.3. Sampling, DNA Extraction and Genotyping

Leaf samples were collected from 108 adult *S. ovata* trees across four natural populations in Wenchang, Hainan, China (Table S1). Sampling was conducted in 2020, with population sizes as follows: DC (48 trees), XC (37 trees), HG (15 trees), and XT (8 trees). Leaves were desiccated in silica gel and stored at −20 °C until DNA extraction.

In December 2020, we collected fruits from 11 mother trees, obtained seeds, and raised seedlings in a greenhouse. The offspring’s leaves were collected separately, and 165 offspring (F1-1~15, F2-1~15, F3-1~15, F4-1~15, F5-1~15, F6-1~15, F7-1~15, F8-1~15, F9-1~15, F10-1~15, F11-1~15) from 11 mother trees were used for SSR analysis. The formal identification of the samples in this study was undertaken by Shi-Quan Wang.

Total genomic DNAs of each mother tree leaves and progeny leaves were extracted using the modified CTAB method [27]. DNA concentration and quality were checked via gel electrophoresis on 1% agarose and NanoDrop One spectrophotomer (Thermo Fisher Scientific, Wilmington, DE, USA). DNA samples were adjusted to working concentration and stored at −20 °C.

Through simplified genome sequencing, there were 274 pairs of markers being selected, and fifteen pairs of markers [28] were used to screen. These primers were tested on representative samples (eight individuals from four populations). Ten pairs of microsatellite primers (Table S2) produced clear, polymorphic, and good amplification effects in all samples and were used for further genotyping.

SSR amplification was performed in a 10 µL reaction system, containing 5 µL of 2×Taq PCR MasterMix (Tiangen Biotech, Beijing, China) [0.1 U/µL Taq DNA Polymerase, 0.5 mM each of combined dNTPs, 20 mM Tris-HCl (pH 8.3), 100 mM KCl, 3 mM MgCl_2_)], 1 µL of DNA template (approximately 20 ng/µL), 3.4 µL double distilled water, and 0.6 μL (10 pmol/µL) of each primer (forward and reverse) with fluorescent-labeled tails (5′-FAM, HEX, TAMRA).

Thermocycler amplifications were executed in a Bio-Rad thermal cycler (Applied Biosystems, Waltham, MA, USA) under the following touchdown program. The first protocol was the amplification of adaptor primers as follows: after an initial 5 min predenaturation step at 95 °C, denaturation 95 °C for 30 s, annealing 62 °C to 52 °C for 30 s running for 10 cycles (with 1 °C decrease in every cycle), denaturation 95 °C for 30 s, annealing 52 °C for 30 s, and extension 72 °C for 30 s running for 25 cycles, and final extension 72 °C for 20 min. The second protocol was amplification of fluorescent primer as follows: after an initial 5 min predenaturation step at 95 °C, denaturation 95 °C for 30 s, annealing 52 °C for 30 s, and extension 72 °C for 30 s running for 25 cycles, and final extension 72 °C for 5 min. PCR products were checked on an ABI 3730xl DNA capillary sequencer (Applied Biosystems, Foster City, CA, USA) with LIZ 500 as an internal size standard. Raw data were obtained and analyzed with GeneMaker version 4.0 (Softgenetics, State College, PA, USA).

### 2.4. Statistical Analysis

Polymorphic information content (*PIC*) was calculated with Cervus 3.0 [29]; allele richness (*A*_r_) and inbreeding coefficient (*F*_is_) were calculated with FSTAT 2.9.3.2 [30]. Basic genetic parameters were computed with GenAlEx 6.5 [31]: number of alleles per locus (*N*_a_), number of effective alleles (*N*_e_), Shannon’s information index (*I*), expected heterozygosity (*H*_e_), observed heterozygosity (*H*_o_), fixation index (*F*), genetic differentiation coefficient (*F*_st_), gene flow (*N*_m_), genetic distance (GD), genetic identity (GI), Mantel test, Hardy–Weinberg equilibrium (HWE), Analysis of molecular variance (AMOVA, No. Permutations: 999), and Principal coordinate analysis (PCoA).

**G**enetic structure was performed by Structure 2.3.4 [32] checking *K* values of 3–9 (1 × 10^5^ MCMC repetitions, after a burn-in length of 1 × 10^5^ iterations under a mixed model with correlated allele frequency). The procedure [33] was used to determine the best *K* value and optimal value of genetic groups (the highest Δ*K*, the best *K* value), which was determined with Structure Harvester V6.0 [34].

Cluster analysis was performed to construct a UPGMA dendrogram with 1000 bootstrap replications through PHYLIP v3.67 [35], according to Nei’s genetic distance (allele frequencies), and was applied to evaluate the genetic relationship between populations.

Based on a mixed mating system model, mating system parameters were calculated with MLTR 3.4 [36] at different levels (population and lineage). Through 1000 bootstraps iterations at a 95% confidence interval, the expectation maximization method was used to estimate the multi-locus outcrossing rate (*t*_m_), single-locus outcrossing rate (*t*_s_), selfing rate (*s*), biparental inbreeding (*t*_m_ − *t*_s_), inbreeding coefficient of single-locus of maternal parents (*F*), number of effective pollen donors (*N*_ep_), and expected inbreeding coefficient (*F*_is_).

## 3. Results

### 3.1. Pollen Viability and Stigma Receptivity

Pollen viability followed a predictable trend, peaking at 89.35% on the third day of flowering (Figure S1A) before decreasing to 40.81% on the fourth day and 18.30% on the 5th day (Table S3). The stigmas remained receptive from the first day to the fourth day (Table S3), strongest on the second to third day of flowering (Figure S1B). The overlap time between pollen viability and stigma receptivity was 4 d.

### 3.2. Mating System

#### 3.2.1. OCI and P/O

OCI was computed through summing the three values below: (a) flower diameter was 9.29 ± 0.14 cm (*n* = 20) > 6 mm, recorded as 3; (b) time separation between stigma receptivity and anther dehiscence: protogyny, recorded as 0; and (c) spatial position between anthers and stigma: spatial separation, recorded as 1. The OCI was 4 accordingly, indicating that the mating system was outcrossing, partially self-compatible, and needed pollinators. The mean single-flower pollen number was 8,651,250 ± 4250.61 and the single-flower ovule number was 3016 ± 163.62, hence P/O was 2955.921 ± 175.04. According to Cruden’s standard P/O of 2108.0-195,525.0 [25], the mating system of *S. ovata* was therefore outcrossing.

#### 3.2.2. Effects of Different Pollination Treatments

The results of different pollination treatments were shown in Table 1. The fruit set rate of seven treatments ranged from 0 to 18.33% and the number of seeds per fruit ranged from 0 to 193.18. In natural pollination, the fruit set rate was low at 11.67%; the fruit set rate of bagging without emasculation was 0, indicating self-crossing sterility and stigma incompatibility with self-pollen; and the fruit set rate of bagging after emasculation was 0, suggesting that there is no apomixis. Netting after emasculation can isolate big insects (bees, moths), resulting in a fruit set rate of 8.33%, suggesting that there is anemophily, but the fruit set rate was lower than that of natural pollination. The single sample *t*-tests for the number of fruit, number of seeds per fruit, and fruit set rate calculated with SPSS v17 were 3.48, 3.66, 3.48, respectively, and all differences were not significant (*p* > 0.05) (Table S4).

#### 3.2.3. Mating System Analysis Based on SSR

The multi-locus outcrossing rate (*t*_m_) was 0.851, single-locus outcrossing rate (*t*_s_) was 0.676, selfing rate (*s*) was 0.149, and biparental inbreeding (*t*_m_ − *t*_s_) was 0.175, showing the existence of a certain proportion of biparental inbreeding. The number of effective pollen donors (*N*_ep_) was 1.025, which further confirmed the above result (Table S5). The single-locus inbreeding coefficient of maternal parents (*F*) was −0.2, which indicated a surplus of heterozygotes and lack of homozygotes, suggesting that the mating system of *S. ovata* is predominantly outcrossing.

Mating systems were analyzed at an individual level for 11 families (Table S6). Variability of the multi-locus outcrossing rate (*t*_m_) and the single-locus outcrossing rate (*t*_s_) among lines was low, with *t*_m_ varying from 0.937 to 1.200 and *t*_s_ from 0.934 to 1.259. Among these 11 families, the maximum value of *t*_m_ − *t*_s_ was 0.109 (family 1), the minimum value was −0.257 (family 9), and the *t*_m_ − *t*_s_ values in families 4, 7, 8, 9, and 10 were less than 0, indicating more heterozygotes and absence of biparental inbreeding.

### 3.3. Genetic Diversity

At the locus and population aspects, the numerical value of genetic diversity parameters (*N*_a_, *N*_e_, *H*_e_, *H*_o_, *I*, *F*) were shown in Table 2 and Table S7. Allele number (*N*_a_) varied from 1.4 (XT) to 3.4 (DC) (mean = 2.375), while 1.5–2.8 (mean = 2.04) in *S. alba* [28]. Effective allele number (*N*_e_) was 1.417, ranging from 1.309 (XT) to 1.523 (HG). Average expected heterozygosity (*H*_e_) and observed heterozygosity (*H*_o_) ranged between 0.143 (XT) and 0.294 (DC) and between 0.152 (XT) and 0.291 (DC), with averages of 0.215 and 0.245, respectively. Meanwhile, *H*e: 0–0.57 (mean = 0.21) and *H*o: 0–0.67 (mean = 0.21) in *S. alba* [28]. Shannon’s information index (*I*) was 0.377, varying from 0.221 (XT) to 0.536 (DC). Fixation index (*F*) averaged −0.051, varying between −0.228 (HG) and 0.096 (DC).

### 3.4. Genetic Differentiation and Gene Flow Between Populations

There was a significant difference in *F*_st_ between populations (*p* < 0.001), ranging between 0.109 (HG and XT) and 0.446 (XC and XT), with an average of 0.256 (*p* < 0.001; Table S8). In contrast, *N*_m_ ranged between 0.311 (XC and XT) and 2.044 (HG and XT), with an average of 0.974 (Table S8).

Nei’s genetic distance was the lowest between HG and XT (0.043) and the highest between XC and XT (0.288) (Table S9). Although the Mantel test suggested a correlation between geographic and genetic distance (*r*^2^ = 0.4841 *p =* 0.05), the relationship was not statistically significant (Figure S2), implying that other factors may influence genetic structure beyond geographic distance. The AMOVA result showed that 75.4% of the molecular variance was within populations and 24.6% was among populations (*p* < 0.001) (Table 3).

### 3.5. Genetic Structure

**Figure 1:**
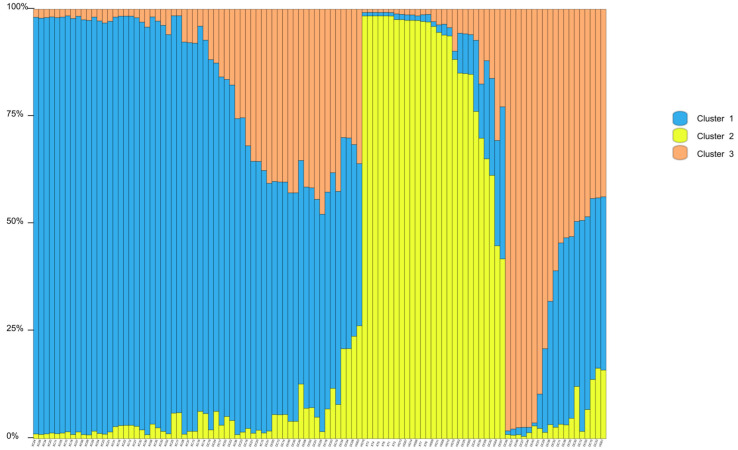
Genetic structure of *Sonneratia ovata* populations inferred via STRUCTURE (*K* = 3).

**Figure 2:**
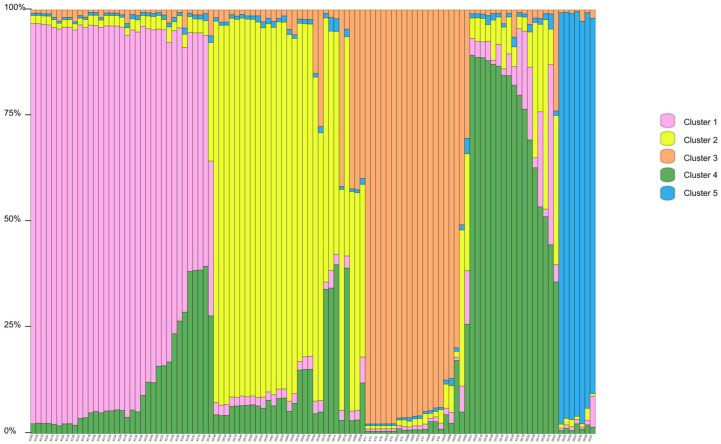
Genetic structure of *Sonneratia ovata* populations inferred via STRUCTURE (*K* = 5).

**Figure 3:**
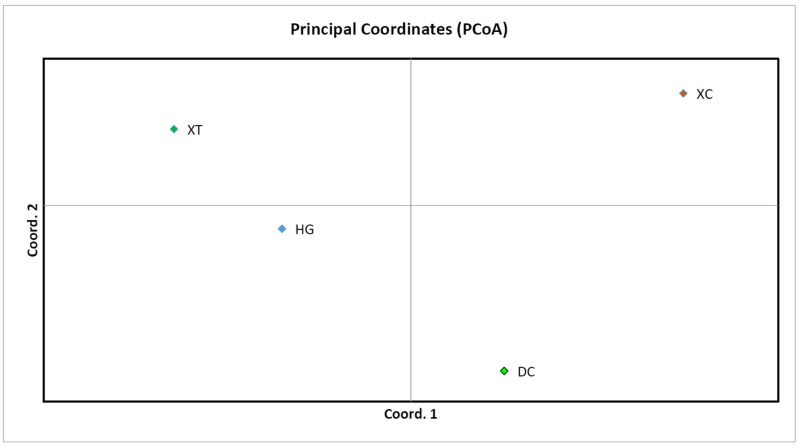
Principal coordinate analysis (PCoA) map of four populations.

Structure Harvester’s Δ*K* plot showed that models with three or five clusters explained the data nearly equally well (the highest Δ*K*, the best *K* value) (Figure S3). Thus, four populations originated from three or five ancestry clusters, and these clusters intermixed (Figure 1 and Figure 2). The first cluster was dominated by individuals from XC, with some individuals from DC and an individual from HG mixed in. In the second cluster, individuals of HG were the main components, mixed with a number of individuals from XT and DC. The third cluster mainly consisted of DC, mixed with some individuals from XC and HG (Figure 1). In the first cluster, individuals of XC were the main components, mixed with an individual from DC. The second cluster was composed of individuals from DC, with some individuals from HG mixed in. The third cluster mainly consisted of individuals from HG, with some individuals from XT and DC. The fourth cluster was made up of individuals from DC, with some individuals from XC. The fifth cluster mainly included individuals from DC and an individual from XC (Figure 2). Principal coordinate analysis (PCoA) presented populations’ genetic structures (Figure 3). Variance percentage owing to three principal coordinate axes was 100% (axis 1—73.70%, axis 2—21.84%, and axis 3—4.46%).

**Figure 4:**
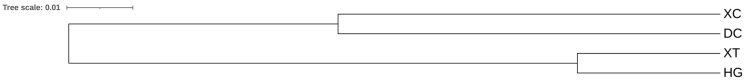
UPGMA tree diagram according to genetic distance.

A UPGMA dendrogram was established based on genetic distance, which reflected the genetic relationship between populations. The UPGMA tree showed that the four populations were split into two groups: 1 and 2 (Figure 4). Group 1 was composed of two populations, namely XC and DC; group 2 consisted of two populations, XT and HG.

## 4. Discussion

### 4.1. Mating System and Pollination Biology

OCI (4), P/O (2955.92), and pollination treatment results show that *S. ovata* has a mixed mating system, with both self- and cross-pollination. The same mixed mating system existed in *S. × hainanensis* (OCI = 4, P/O = 354) [22]. The biparental inbreeding values (*t*_m_ − *t*_s_ = 0.175) implied that a certain degree of inbreeding existed within the population. There are many mangrove plants (*S. alba*, *S. caseolaris*, *S × gulngai*, *S × hainanensis*, *Lumnitzera racemosa*, *Avicennia marina*, *Scyphiphora hydroplyllacea*, *Xylocarpus granatum*) that bloom at the same time, and they compete for pollinators with *S. ovata*, resulting in pollinator restrictions.

The mixed mating system indicates an adaptation for the survival of *S. ovata* in nature, through which populations may be kept stable, in order to compensate for the lack of pollinators. The populations of the endangered plant *S. ovata* are small, fragmented, and isolated, and the number of individuals is limited. Selfing is expected to decrease heterozygosity, resulting in the expression of deleterious recessive alleles and an increase in inbreeding depression [37].

### 4.2. Genetic Diversity and Population Structure

In general, there is low genetic diversity in endangered species, whose distribution is narrow compared with widespread species [38]. Because of their small population numbers and isolated populations from each other, they have adapted to specific habitats [39].

Genetic diversity of *S. ovata* (*H*_e_ = 0.215) is lower than that of endemic species (*H*_e_ = 0.420) and widely distributed species (*H*_e_ = 0.620) [40]. Compared with previous research using ISSRs, genetic diversity parameters here were somewhat higher than those of *S. hainanensis* (*h* (expected heterozygosity) = 0.1538) [13], *S. caseolaris* (*h* = 0.1468) [14], *S. ovata* (*h* = 0.1209) [15], *S. alba* (*h* = 0.1837) [16], and *S. apetala* (*H*_e_ = 0.1403) [17], because SSR had more advantages (co-dominant, highly polymorphic, highly reproducible, highly reliable) [19] than ISSRs.

Genetic diversity was higher in population DC than that in other populations, because this population is large and little affected by people. Perhaps here is a possible historical refugium. Thus, DC may represent the center of genetic diversity. Fixation index (*F*) showed that two populations (HG, XT: negative value) revealed excessive heterozygotes, suggesting outbreeding. However, the other two populations (positive value) had excessive homozygotes, suggesting inbreeding. The average negative inbreeding coefficient (*F*_is_) value (−0.112) showed excessive heterozygotes in *S. ovata* (Table 2).

The UPGMA classified the four populations into two groups, showing two distinct genetic clusters, which resulted from geographic separation and limited gene flow. Structure analysis obviously manifested three or five ancestry clusters, and some samples were admixed. The admixture showed that gene flow still occurred in some populations (supported by higher *N*_m_ values between populations). Sea flow is the ecological or geographical factors facilitating this gene flow, despite fragmentation. The fruits and seeds can be moved from one place to another as the sea tide rises and falls. Pollen flow may happen between populations because of pollination by animals. In addition, the PCoA result was identical to the UPGMA cluster result.

The genetic relationship between populations reflected the natural geographical areas of populations, which was supported by the isolation-by-distance (IBD) model drawn through the Mantel test. The IBD model showed positive correlation (*r*^2^ = 0.4841, *p* = 0.05) between geographic and genetic distance. Due to the seawater environment in which *S. ovata* lives, seawater flow may affect genetic distance, gene flow, and genetic differentiation, resulting in a weak positive correlation between geographic and genetic distance.

### 4.3. Gene Flow and Genetic Differentiation

The relatively high gene flow between HG and XT (*N*_m_ = 2.044) suggests genetic connectivity. In contrast, the restricted gene flow between XC and XT (*N*_m_ = 0.311) may indicate significant geographic distance, geographic barriers, or habitat fragmentation, which could contribute to population divergence.

Gene flow (*N*_m_), as a fundamental micro-evolutionary phenomenon, impedes genetic differentiation between populations and influences genetic diversity sustenance [41]. Numerous endangered species are separated and narrowly distributed as petty populations, perhaps residual of formerly large continuous widespread populations [42]. Genetic drift will result in substantial local differentiation if *N*_m_ < 1 but not if *N*_m_ > 1 [43]. Gene flow (>1) between populations (HG and XT, HG and DC) (Table S8) prevented genetic differentiation as a result of genetic drift. Genetic drift was not the primary factor affecting genetic structure. Most of the genetic differentiation was high, except for the moderate level between HG and XT (Table S8), based on suggestions by Wright [44].

AMOVA results showed that molecular variance mainly occurred intra-populations as opposed to inter-populations, which was the same as other studies using ISSR markers [14,16,17]. Generally, major genetic variation exists intra-populations of outcrossing and long-lived plants. However, major genetic variation occurs inter-populations of selfing plants [40].

Due to aquaculture, tourism, raising poultry, water pollution, etc., habitats have been seriously destroyed and deteriorated, which results in habitat fragmentation. Habitat fragmentation mainly affects plant genetic diversity by influencing gene exchange. Habitat fragmentation can lead to an increase in inter-patch distance, which not only reduces plant population connectivity and affects population abundance, but also affects the pollination behavior of pollinators, thereby limiting the spread of plant pollen and seeds [45,46]. Therefore, even with long-distance gene exchange mediated by wind, insect vectors, and sea flow (this study), genetic diversity can still be affected by habitat fragmentation [47], leading to a high outcrossing rate with low genetic diversity in *S. ovata*.

### 4.4. Conservation Implications and Management Strategies

Genetic variation in intra- and inter- populations in endangered species plays an important part during the process of enacting a protection and management strategy [48]. Considering the rapid decrease in population numbers and the destruction of natural habitats, it is imperative to take action for in situ and ex situ conservation. Populations with high genetic diversity or genetic difference should be protected as a priority. In situ conservation is regarded as the most effective way to protect endangered species, because the total genetic bank can be conserved in the natural habitat. Small populations are more easily extinct because of habitat destruction. It is crucial to protect all individuals and populations in situ in order to preserve possible genetic variation. Population DC, with higher genetic diversity compared with other populations, should be conserved as a priority. Some research showed that heterozygosity could guarantee the adaptation fitness and potential of populations [49]. However, the lack of heterozygotes in DC and XT maybe resulted from inbreeding in fragmented populations.

Conservation is focused on preserving four populations. DC, with greater genetic diversity, should have specific aims for ex situ conservation. Because most of the genetic differentiation was high between populations, every differentiation represented an important component of genetic variation. Therefore, seed collection from all populations should be planned to construct a germplasm resource bank ex situ for gathering more seeds, and conserve germplasm via tissue culture techniques. In the process of ex situ conservation, artificial hybridization should be executed between populations to enhance heterozygosity. In short, population conservation in situ and ex situ must be integrated to conserve worthy germplasm resources. In addition, habitat restoration, control of anthropogenic threats, pollination management, avoidance of crossing highly divergent pairs, and community involvement should be considered so as to guarantee a more holistic conservation plan. Because of the limitation of the number of samples, populations, and loci, and the lack of research on human activities and environmental factors, it is necessary to increase relevant research (human, environment, habitat factors, etc.) to better protect and utilize this species.

## 5. Conclusions

This study provides reproductive traits through pollination investigation and SSR marker experiment in *S. ovata*. It is a hermaphroditic species, with a mixed mating system, partial self-compatibility, and needs pollinators. *S. ovata* does not reproduce via apomixis but shows anemophily and experiences inbreeding depression. Natural populations maintained high outcrossing coupled with inbreeding and low genetic diversity. The population DC was regarded as the center of genetic diversity in *S. ovata*. Four populations classified into two groups were regarded as two conservation and management units. They are valuable germplasm resources for breeding plans and conservation strategies in the future. This study is the first to investigate the reproductive traits, genetic diversity, and population structure of *S. ovata* via SSR. The results provide a scientific basis for cross breeding, conservation, and management.

The markers employed in this study can be used to investigate germplasm collection and conservation strategies. The markers supply prominent information on genetic structure, which is obviously conducive to improvement and breeding planning in the future. The genetic diversity, population structure, and genetic relationship among populations based on SSR will be useful for plant breeding, idioplasm management, and species conservation.

## Figures and Tables

**Table 1:** Effects of different pollination treatments on fruiting status of *Sonneratia ovata*.

Pollination Treatments	Number of Treated Flowers	Number of Fruits	Number of Seeds Per Fruit	Fruit Set Rate (%)
Nature pollination	60	7	172.29 ± 40.30	11.67
Bagging without emasculation	60	0	0	0.00
Bagging after emasculation	60	0	0	0.00
Netting after emasculation	60	5	107.20 ± 19.17	8.33
Artificial geitonogamy	60	10	170.64 ± 23.87	16.67
Artificial xenogamy	60	11	191.28 ± 29.63	18.33
Hand supplementary pollination	60	10	193.18 ± 19.31	16.67

**Table 2:** Genetic parameters of four populations of *Sonneratia ovata*.

Population ID	*N*_a_	*N*_e_	*I*	*H*_o_	*H*_e_	*F*	*PIC*	*A*_r_	*PPL*	*F*_is_	*HWE*
DC	3.400	1.512	0.536	0.291	0.294	0.096	0.259	2.203	100.00%	0.022	SO24 ***, SO67 ***, SO77 ***
HG	2.200	1.523	0.386	0.284	0.218	−0.228	0.191	1.884	60.00%	−0.271	SO52 **, SO77 **, SO177 *
XT	1.400	1.309	0.221	0.152	0.143	−0.153	0.119	1.400	30.00%	0.012	SO77 **, SO177 *
XC	2.500	1.324	0.363	0.253	0.206	0.083	0.178	1.797	90.00%	−0.210	SO24 ***, SO67 ***, SO77 ***, SO177 *
mean	2.375	1.417	0.377	0.245	0.215	−0.051	0.187	1.821	70.00%	−0.112	

*N*_a_: The observed number of alleles. *N*_e_: The effective number of alleles. *I*: Shannon’s information index. *H*_o_: Observed heterozygosity. *H*_e_: Expected heterozygosity. *F*: Fixation index. *PIC*: Polymorphism information content. *A*_r_: Allelic richness. *PPL*: the percentage of polymorphic loci. *F*_is_: Inbreeding coefficient among individuals within populations. HWE: loci showing a significant departure from Hardy–Weinberg equilibrium with a global test at 5% level and after a sequential Bonferroni correction (* *p* < 0.05. ** *p* < 0.01. *** *p* < 0.001).

**Table 3:** Analysis of molecular variance (AMOVA) for four populations of *Sonneratia ovata*.

Source of Variance	Degree of Freedom	Sum of Squares	Variance Components	Total Variance (%)	*p*-Value
Among populations	3	71.080	0.468	24.60	<0.001
Among individuals	104	165.355	0.165	8.75	<0.001
Within individuals	108	136.000	1.259	66.65	<0.001
Total	215	372.435		100%	

## Data Availability

The datasets generated and/or analyzed during the current study are available in the Genome Sequence Archive (GSA) repository, https://ngdc.cncb.ac.cn/omix/select-edit/OMIX009131 (accessed on 20 February 2025) (BioProject: PRJCA036255).

## Supplementary Materials

The following supporting information can be downloaded at https://www.mdpi.com/article/10.3390/biology14111580/s1, Table S1: Sample information from 4 populations of *Sonneratia ovata*; Table S2: Characteristics of 10 polymorphic SSR primers; Table S3: Pollen viability and stigma receptivity of *Sonneratia ovata*; Table S4: One-sample *t* test in number of fruit, number of seeds per fruit, fruit set rate in *Sonneratia ovata*; Table S5: Parameter of mating system of *Sonneratia ovata* based on population level; Table S6: Parameter of mating system of *Sonneratia ovata* based on individual level; Table S7: Genetic diversity parameter of loci based on 108 individuals of *Sonneratia ovata*; Table S8: Genetic differentiation coefficient (*F*_st_) (below diagonal) and gene flow (*N*_m_) (above diagonal) between populations; Table S9: Nei’s genetic distance (below diagonal) and genetic identity (above diagonal) of 4 populations. Bold character indicates the highest value, while italic character displays the lowest value; Figure S1: Pollen viability by sucrose boronic-acid germination method (A) and stigma receptivity by benzidine-hydrogen peroxide method (B) of *S. ovata*; Figure S2: Correlation map of genetic distance (GD) and geographic distance (GGD); Figure S3: The distribution of ΔK over K = 3~9.

## Abbreviations

AP-PCR	Arbitrarily primed PCR
FAA	Formaldehyde, acetic acid, alcohol
AMOVA	Analysis of molecular variance
HWE	Hardy–Weinberg equilibrium
IBD	Isolation-by-distance
ISSR	Inter simple sequence repeat
OCI	Outcrossing Index
P/O	Pollen–ovule ratio
PcoA	Principal coordinate analysis
RAPD	Random amplified polymorphic DNA
SSR	Simple sequence repeat
OCIUPGMA	unweighted pair group method with arithmetic mean
