# Supplementary material for: Reproductive Biology and Population Structure of the Endangered Species Sonneratia ovata Backer
Source: Biology (Basel). 2025 Nov 12;14(11):1580. doi: 10.3390/biology14111580 (PMC12649928; doi:10.3390/biology14111580)
Supplement: Supplementary file 1 [file biology-14-01580-s001.zip › biology-3876293-supplementary.pdf]

Table S1. Sample information from 4 populations of *Sonneratia ovata*.

Population	Population code	Geographical coordinate		Sample capacity
		Latitude (N)	Longitude (E)	
Danchan	DC	19°37'32.718"	110°48'51.768"	48
Xiachang	XC	19°37'02.842"	110°47'52.084"	37
Hougang	HG	19°37'16.147"	110°48'37.494"	15
Xitou	XT	19°37'08.382"	110°52'31.307"	8

Table S2. Characteristics of 10 polymorphic SSR primers.

Locus	Primer sequence (5′–3′)	Primer sequence (3′–5′)	Repeat motif
SO24	ACGGTCAAGTTCTGGTGTCC	AGTCACGGGAAGAGTCATGG	$(TGA)_7$
SO52	ACTGCCTCTCATTCCCACAC	GTTTGTGTCGTCGAGGGTTT	$(TA)_8$
SO67	GAAGGAAGAGGAAACCGGAC	CGGGTTGAATTTAAGCTGGA	$(GA)_{10}$
SO71	TTTGTTCAGCCCTACCTTGG	GCAGTGCTCTTGCATCATGT	$(CT)_{13}$
SO77	TACGGCTCTATGGCTTGCTT	TGTGAAGTGTCTTGCTTGGC	$(AG)_7$
SO146	ACAATCCGTCTATGCCTTGC	CGAACACTCTCTCGACTCCC	$(GA)_{17}$
SO177	AGCGTGTACGACAAAGCAGA	CACGACAATTAGGGGTTGCT	$(GA)_{15}$
SO195	CGTGGGCAAAACTAAACACA	TAGCGGAAAATCTCTCCCTC	$(AG)_{15}$
SA112 [28]	ATGATTGAGTCCGTTCCGTC	GTTTAGCAGAGCAGTCCACTCGAT	$(AG)_{11}$
SA117 [28]	CCTCGAAGACGCAGTAAACC	GTTTCGACGACAAGTGTGAAAGG	$(AGG)_{11}$

Table S3. Pollen viability and stigma receptivity of *Sonneratia ovata*.

Flowering time	Pollen viability (%)	Stigma receptivity
Day 1	63.22±6.39	+
Day 2	85.15±4.95	++
Day 3	89.35±2.25	++

Day 4	40.81±8.04	+/−
Day 5	18.30±5.46	−

Note: −, means no stigma receptivity; +, means stigmas have receptivity; ++, means stigmas have high receptivity.

Table S4. One-sample *t* test in number of fruit, number of seeds per fruit, fruit set rate in *Sonneratia ovata*.

Traits	*t*	df	Sig. (2-tailed)	Mean diference	95% confdence interval of the diference	
					Lower	Upper
Number of fruit	3.48	6	0.013	6.143	1.824	10.462
Number of seeds per fruit	3.66	6	0.011	119.227	39.406	199.049
Fruit set rate	3.48	6	0.013	10.239	3.0396	17.438

Table S5. Parameter of mating system of *Sonneratia ovata* based on population level.

Parameter	Estimates (95%CI)
Number of families (total number of progenies)	11 (165)
Multi-locus outcrossing rate (t_m)	0.851 (0.099)
Single-locus outcrossing rate (t_s)	0.676 (0.085)
Selfing rate (s)	0.149
Biparental inbreeding (t_m-t_s)	0.175 (0.049)
Multi-locus correlation of paternity ($r_{p(m)}$)	0.976 (0.125)
Single-locus correlation of paternity ($r_{p(s)}$)	0.999 (0.162)
Single-locus inbreeding coefficient of maternal parents (F)	-0.200 (0.041)
Number of effective pollen donors (N_{ep})	1.025
Expected inbreeding coefficient (F_e)	0.080

Table S6. Parameter of mating system of *Sonneratia ovata* based on individual level.

Family code	Total number of progenies	t_m	t_s	t_m-t_s
F1	15	1.200 (0.512)	1.091 (0.479)	0.109 (0.051)
F2	15	1.131 (0.497)	1.063 (0.463)	0.068 (0.044)
F3	15	1.000 (0.001)	0.934 (0.051)	0.066 (0.001)
F4	15	1.162 (0.501)	1.200 (0.512)	-0.038 (0.019)
F5	15	1.200 (0.512)	1.133 (0.493)	0.067 (0.033)
F6	15	1.200 (0.000)	1.196 (0.002)	0.004 (0.002)
F7	15	1.002 (0.001)	1.120 (0.044)	-0.118 (0.056)
F8	15	0.937 (0.447)	0.987 (0.457)	-0.049 (0.024)
F9	15	1.002 (0.001)	1.259 (0.066)	-0.257 (0.002)
F10	15	1.190 (0.462)	1.200 (0.463)	-0.010 (0.102)
F11	15	1.200 (0.512)	1.180 (0.507)	0.020 (0.010)

Table S7. Genetic diversity parameter of loci based on 108 individuals of *Sonneratia ovata*.

Locus	N_a	N_e	I	H_o	H_e	F	PIC	A_r	F_{is}	F_{st}	N_m
SO24	2	1.138	0.241	0	0.122	1	0.114	1.620	1	0.072	3.207
SO52	4	1.743	0.770	0.286	0.426	0.330	0.383	2.737	-0.219	0.469	0.283
SO67	2	1.138	0.241	0	0.122	1	0.114	1.624	1	0.074	3.116
SO71	5	1.333	0.536	0.250	0.250	0	0.237	2.373	-0.054	0.086	2.653
SO77	2	1.990	0.691	0.929	0.498	-0.868	0.374	2	-0.900	0.002	122.909
SO146	8	1.078	0.238	0.074	0.072	-0.023	0.072	1.515	-0.030	0.013	19.237
SO177	6	1.693	0.858	0.308	0.409	0.247	0.381	3.097	0.284	0.211	0.935
SO195	7	1.966	0.975	0.433	0.491	0.119	0.441	3.164	-0.007	0.181	1.133
SA112	2	1.327	0.412	0.287	0.247	-0.164	0.216	1.894	-0.182	0.051	4.658
SA117	2	1.927	0.674	0.102	0.481	0.788	0.365	1.999	0.0210	0.856	0.042

mean	4	1.533	0.564	0.267	0.312	0.243	0.270	2.202	0.091	0.202	15.817

N_a: The observed number of alleles; N_e: The effective number of alleles; I: Shannon's information index; H_o: Observed heterozygosity; H_e: Expected heterozygosity; F: Fixation index; *PIC*: Polymorphism information content; A_r: Allelic richness; F_{is}: Inbreeding coefficient among individuals within populations; F_{st}: Average genetic differentiation coefficient; N_m: Gene flow.

Table S8. Genetic differentiation coefficient (F_{st}) (below diagonal) and gene flow (N_m) (above diagonal) between populations. Bold character indicates the highest value, while italic character displays the lowest value.

	DC	HG	XT	XC
DC	–	1.392	0.682	0.950
HG	0.152	–	**2.044**	0.463
XT	0.268	***0.109***	–	***0.311***
XC	0.208	0.350	**0.446**	–

Table S9. Nei's genetic distance (below diagonal) and genetic identity (above diagonal) of 4 populations. Bold character indicates the highest value, while italic character displays the lowest value.

Nei's Genetic Distance vs Nei's Genetic Identity	DC	HG	XT	XC
DC	–	0.901	0.828	0.891
HG	0.104	–	**0.958**	0.811
XT	0.189	***0.043***	–	***0.750***
XC	0.116	0.209	**0.288**	–

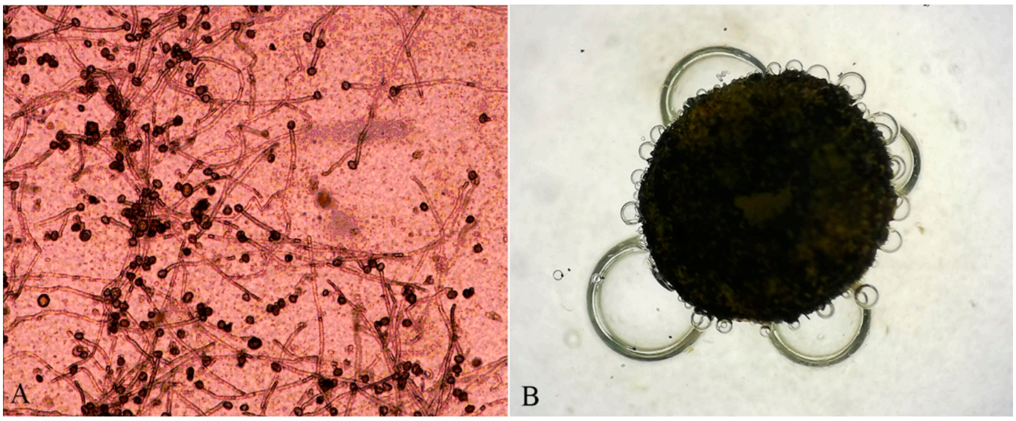

Figure S1. Pollen viability by sucrose boronic-acid germination method (A) and stigma

receptivity by benzidine-hydrogen peroxide method (B) of *S. ovata*

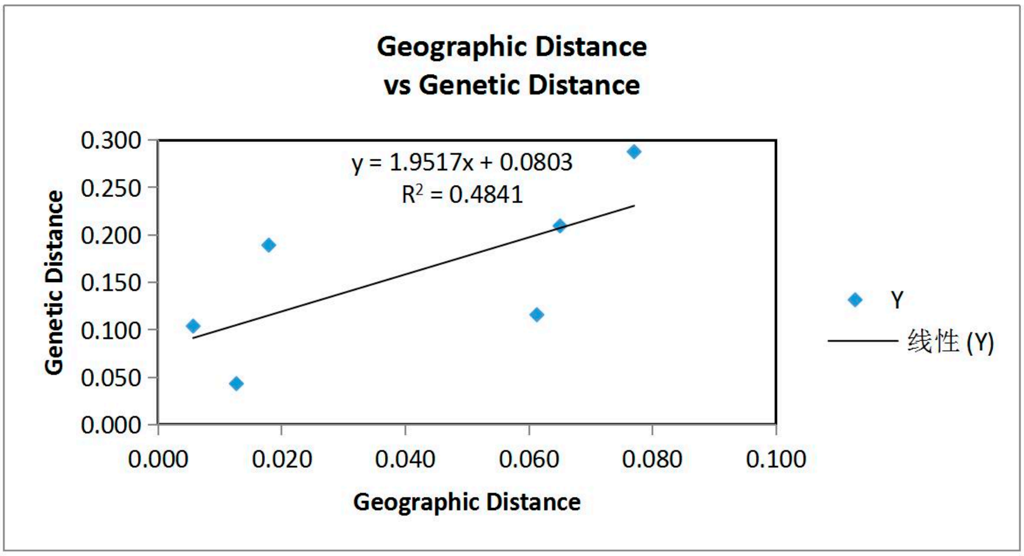

Figure S2. Correlation map of genetic distance (GD) and geographic distance (GGD).

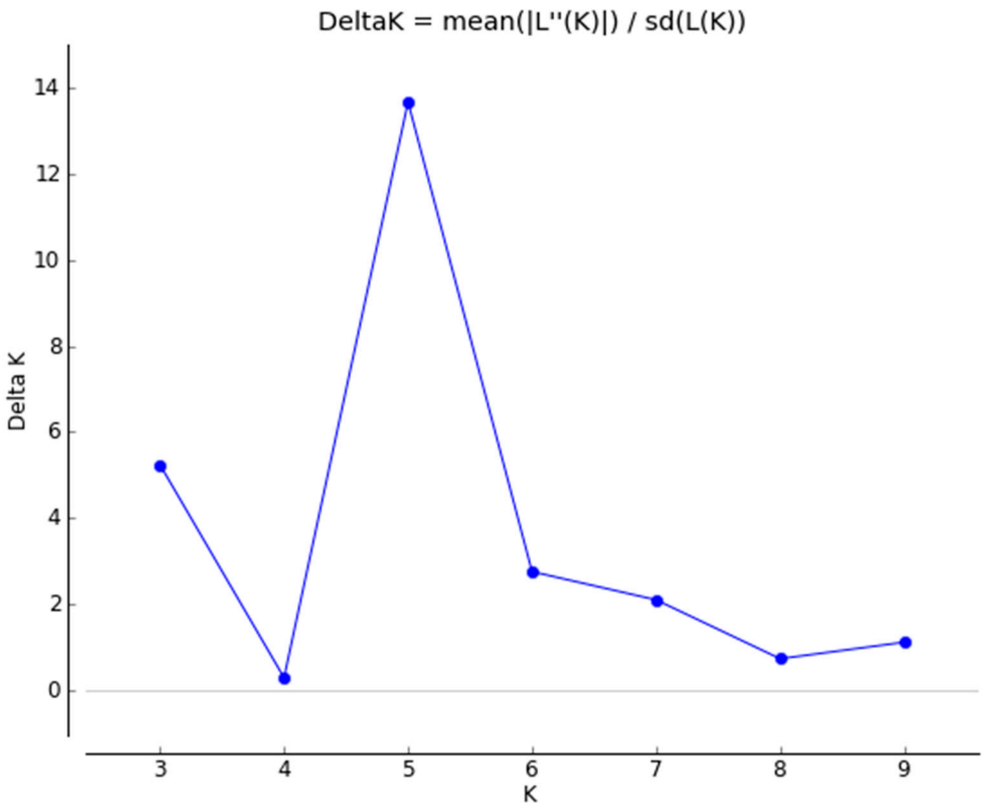

Figure S3. The distribution of ΔK over K=3–9.
